# Non-invasive imaging of disrupted protein homeostasis induced by proteasome inhibitor treatment using chemical exchange saturation transfer MRI

**DOI:** 10.1038/s41598-018-33549-x

**Published:** 2018-10-10

**Authors:** Yanan Zhu, Rajiv Ramasawmy, Sean Peter Johnson, Valerie Taylor, Alasdair Gibb, R. Barbara Pedley, Nibedita Chattopadhyay, Mark F. Lythgoe, Xavier Golay, Daniel Bradley, Simon Walker-Samuel

**Affiliations:** 1 Centre for Advanced Biomedical Imaging, Division of Medicine, University College London, London, WC1E 6DD United Kingdom; 2 Neuroscience, Physiology & Pharmacology, University College London, London, WC1E 6BT United Kingdom; 3 Cancer Institute, University College London, London, WC1E 6DD United Kingdom; 4 Cancer Pharmacology, Takeda Pharmaceutical International Corporation, Cambridge, MA 02139 United States; 5 Institute of Neurology, University College London, London, WC1N 3BG United Kingdom; 6 Biomedical Imaging Group, Takeda Pharmaceutical International Corporation, Cambridge, MA 02139 United States

## Abstract

Proteasome inhibitors (PIs) are now standard of care for several cancers, and noninvasive biomarkers of treatment response are critically required for early patient stratification and treatment personalization. The present study evaluated whether chemical exchange (CEST) magnetic resonance imaging (MRI) can provide measurements that can be used as the noninvasive biomarkers of proteasome inhibition, alongside diffusion MRI and relaxometry. The sensitivity of human colorectal carcinoma cells to the PI Ixazomib was assessed via *in vitro* and *in vivo* dose-response experiments. Acute *in vivo* response to Ixazomib was assessed at three dosing concentrations, using CEST MRI (amide, amine, hydroxyl signals), diffusion MRI (ADC) and relaxometry (T_1_, T_2_). These responses were further evaluated with the known histological markers for Ixazomib and Bradford assay *ex vivo*. The CEST signal from amides and amines increased in proportion to Ixazomib dose in colorectal cancer xenografts. The cell lines differed in their sensitivity to Ixazomib, which was reflected in the MRI measurements. A mild stimulation in tumor growth was observed at low Ixazomib doses. Our results identify CEST MRI as a promising method for safely and noninvasively monitoring disrupted tumor protein homeostasis induced by proteasome inhibitor treatment, and for stratifying sensitivity between tumor types.

## Introduction

Loss of protein homeostasis is associated with a range of pathological conditions including most forms of dementia^1^, amyloidosis^2^ and cancers^3^. In cancer, malignant cells exhibit an abnormally high turnover of protein, which is most notably exploited as a therapeutic target by proteasome inhibitors (PIs)^4^. By inhibiting the action of proteasomes, PIs disrupt protein homeostasis, resulting in apoptotic cell death^5^. PIs are now in routine clinical use for treating hematological malignancies^6–8^, and there is increasing interest in the use of proteasome inhibition as a therapeutic target in solid tumors^9–14^.

The translation of novel therapies into the clinic requires noninvasive biomarkers of successful response to therapy, to enable safe, longitudinal monitoring of disease progression. Assessing response to Pl therapy is currently evaluated invasively (via immunohistochemistry of biopsied tumor tissue)^15^, or radiologically with longitudinal measurements of tumor size^16^. However, such changes in tumor volume can take weeks or months to occur, and so biomarkers that provide a much earlier measurement of response would be extremely valuable. Here, we propose a new set of noninvasive imaging biomarkers for detecting disrupted protein homeostasis, resulting from PI therapy, which are based on magnetic resonance imaging (MRI). In particular, we have focused on chemical exchange saturation transfer (CEST) MRI, which has previously been used to detect changes in protein content in cancer^17–19^. CEST image contrast can be tuned to reflect the exchange of protons between water and various chemical groups, including amides (in protein backbones), amines, guanidinium and hydroxyls^20^. This enables the small signal from solutes containing such groups to be amplified via the much larger water pool signal. We hypothesized that successful proteasome inhibition, and associated protein accumulation^4,21^, would result in an increase in CEST signal of amide and amine chemical groups.

Alongside CEST, we evaluated a range of quantitative, noninvasive MRI measurements (diffusion MRI, *T*_1_ and *T*_2_ relaxometry), in LS174T and SW1222 xenograft models of human colorectal carcinoma. *In vivo* measurements of acute (up to 72 hours) dosing with Ixazomib, at three different dose concentrations, were undertaken and compared with gold-standard histological measures^5,22,23^. Biological validation studies, such as undertaken here, are a vital component of the roadmap for the translation of imaging biomarkers into the clinic^24^. Our study focuses on Ixazomib (Ninlaro®/MLN2238/MLN9708, Takeda Pharmaceuticals Company Limited), which is the first orally-available PI therapy^10,25^ with approval for use in the United States, Europe and Japan for combinational treatment in multiple myeloma^25,26^. Ixazomib has also shown promise in solid tumor rodent models, including colorectal tumors^9–14^.

The aims of our study were to: (1) determine the efficacy of Ixazomib in colorectal carcinoma cell lines and murine xenografts; and (2) evaluate the ability of noninvasive MRI measurements to evaluate this response *in vivo*, with a view to their translation into the clinic.

## Results

### LS174T and SW1222 human colorectal cancer cell lines displayed differing *in vitro* sensitivities to Ixazomib

**Figure 1:**
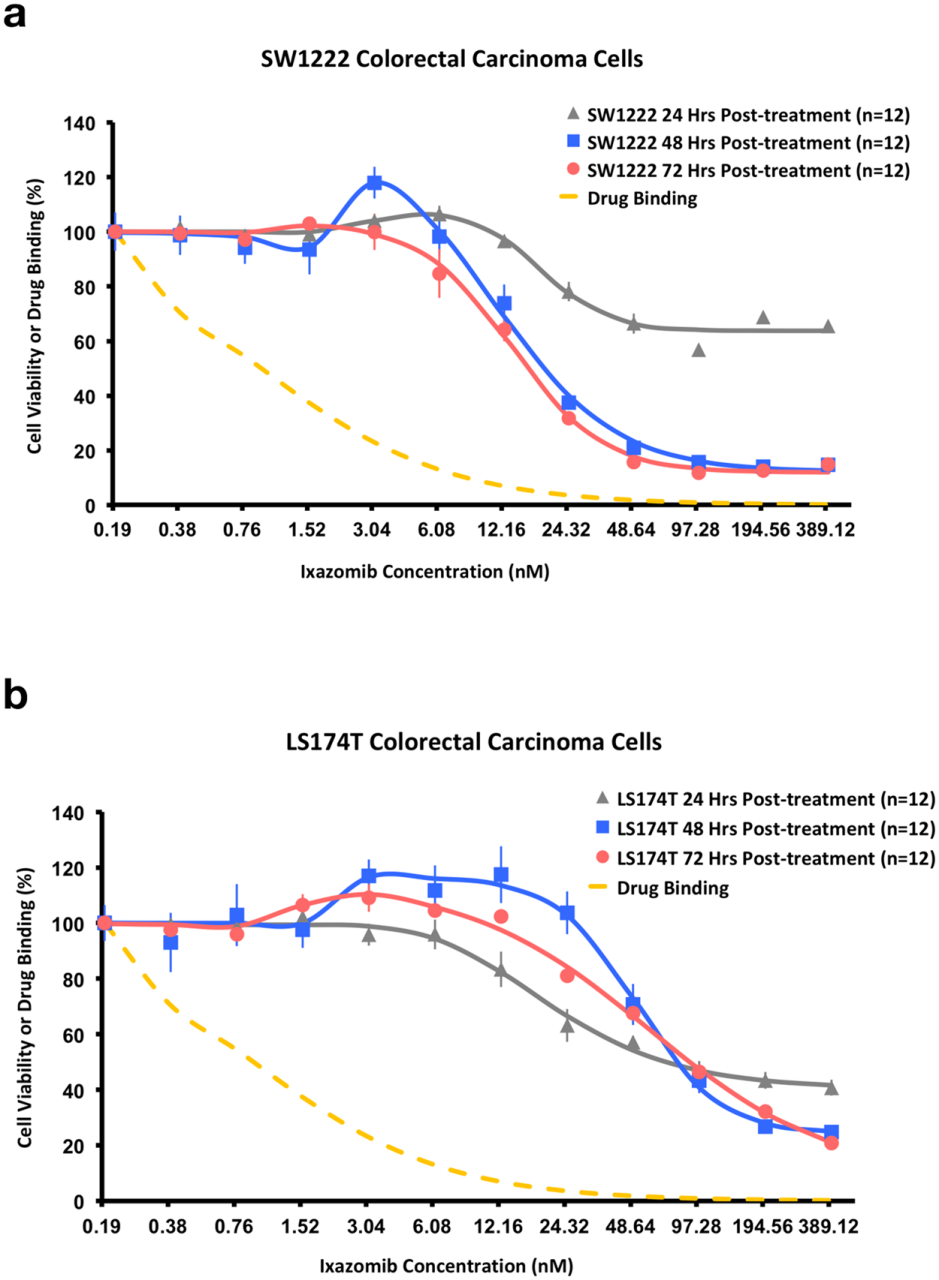
Dose-response curves showing cell viability as a function of Ixazomib concentration, measured with the MTT assay, at 24, 48 and 72 hours post-dosing, in SW1222 (n = 12) (**a**) and LS174T (n = 12) (**b**) cells (data points are mean ± standard error). These *in vitro* results show that cell viability decreases at higher Ixazomib doses (>12 nM in SW1222 and >24 nM in LS174T) and that growth is stimulated at lower doses (<6 nM in SW1222 and <24 nM in LS174T). SW1222 cells are more sensitive to Ixazomib than LS174T cells, which is in keeping with their K-Ras mutation status. Dashed lines show the Ixazomib binding estimates, which is a measure of the percentage of receptors that remain unbound (using (*K*_*i*_ = 0.93 nM, as a function of concentration; as concentration increases, the greater the percentage saturation of binding sites^5^). Data points are mean ± standard error.

The MTT (3-(4,5-Dimethylthiazol-2-yl)-2,5-Diphenyltetrazolium Bromide) assay showed that both SW1222 and LS174T cell lines demonstrated a significant decrease in viability at doses greater than 12 nM in SW1222 and 24 nM in LS174T cells (Fig. 1), at 24, 48 and 72 hours. Interestingly, both cell lines also exhibited a stimulatory response at low doses (<6 nM in SW1222 and <24 nM in LS174T), evidenced by an increase in cell viability (130% in SW1222 and 120% in LS174T) that peaked at 48 hours. Overall, SW1222 cells were more sensitive to Ixazomib than LS174T cells (*IC*_50_ of 12.6 nM and 41.1 nM for SW1222 and LS174T, respectively), with an approximately three-fold lower viability at 48 hours, and approximately ten-fold lower viability at 72 hours (*IC*_50_ of 7.6 nM and 78.4 nM for SW1222 and LS174T, respectively).

### Ixazomib affected colorectal tumor xenograft growth *in vivo*, in a dose-dependent manner

**Figure 2:**
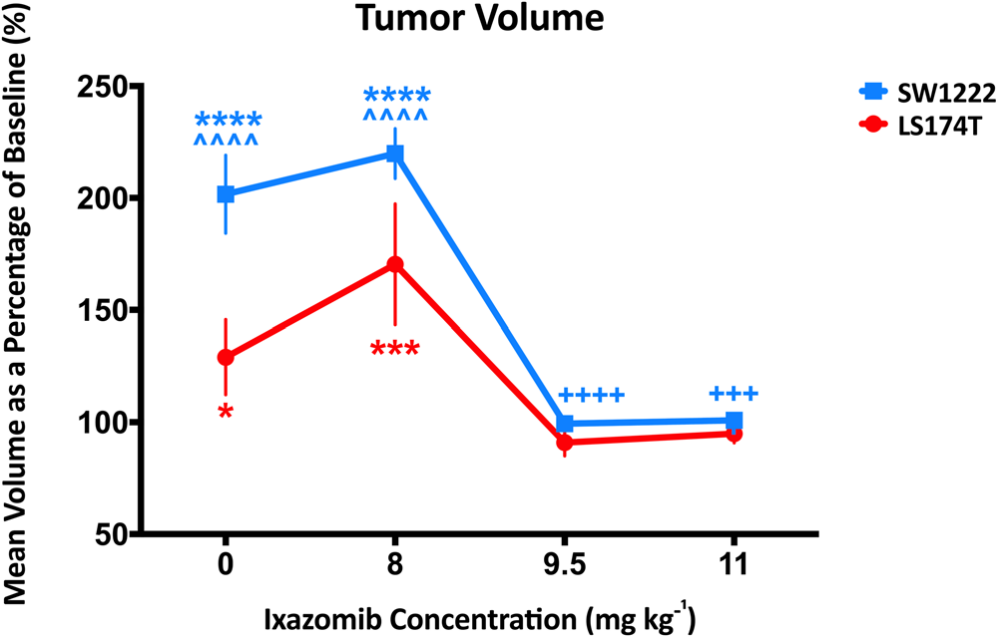
Mean relative change in tumor volume as a function of Ixazomib dose, at 72 hours post-dosing, in LS174T and SW1222 human colorectal carcinoma mouse xenografts. Tumor growth was significantly inhibited at the two highest doses investigated (9.5 and 11 mg kg^−1^), and mildly (but significantly) stimulated at the lowest dose (8 mg kg^−1^). Tumor volumes were measured using volumetric MRI, and error bars represent the standard error in each measurement; *P < 0.05, **P < 0.01, ***P < 0.001, ****P < 0.0001 (two-way ANOVA); *compared to baseline (pre-dosing) measurement, ^^^compared to measurement at 24 hours after Ixazomib dose, ^+^compared to control measurement.

We next investigated the action of Ixazomib *in vivo*, at 24 and 72 hours after dosing with Ixazomib (at 11, 9.5 or 8 mg kg^−1^) or vehicle. MRI measurements of tumor volume confirmed that the growth of both SW1222 and LS174T tumors was significantly inhibited following Ixazomib doses of 9.5 and 11 mg kg^−1^ relative to control tumors (Figs 2 and S1). We also observed a small but significant increase in tumor volume at 72 hours (relative to baseline) at the lowest Ixazomib dose (8 mg kg^−1^) in both SW1222 (P < 0.0001) and LS174T (P < 0.001) tumors, which potentially mirrors the low-dose stimulatory effect that were observed in *in vitro* experiments.

### CEST MRI measurements reflected response to proteasome inhibition in colorectal cancer xenografts

**Figure 3:**
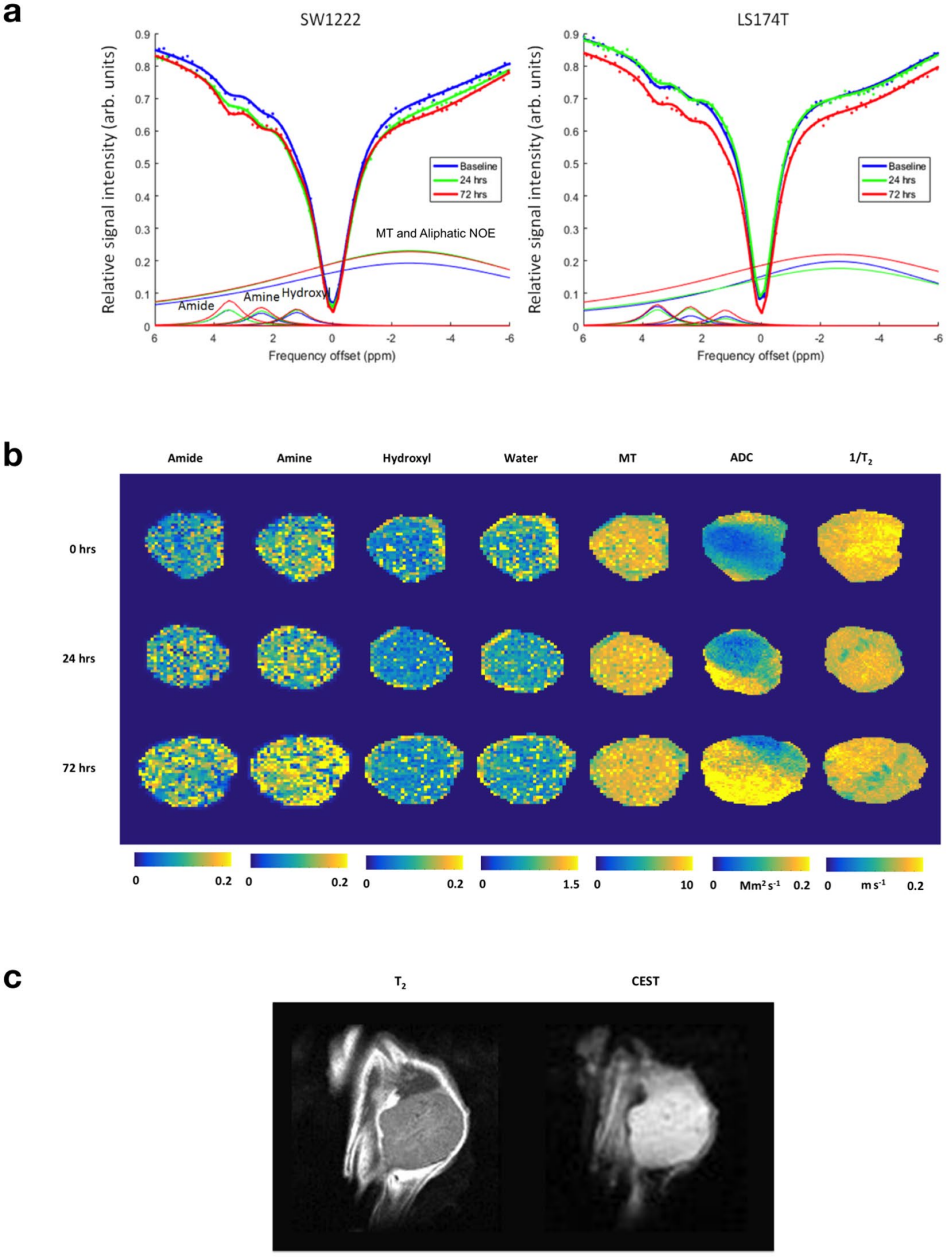
Example MRI measurements in SW1222 and LS174T tumors, prior to and at 24 and 72 hours following treatment with Ixazomib, showing increasing amide and amine peak areas, alongside increasing apparent diffusion coefficient (ADC). (**a**) Z-spectra from CEST MRI, showing the normalised MRI signal intensity as a function of saturation frequency offset, averaged over a single representative tumor dataset at baseline and 24 and 72 hours following dosing with 11 mg kg^−1^ of Ixazomib. Data points were fitted to a Bayesian model that allowed the individual influence of proton exchange between water and amide, amine and hydroxyl groups to be isolated (1.2, 2.4 and 3.5 ppm, respectively), alongside the combined effect of magnetisation transfer (MT, −2.4 ppm) and nuclear Overhauser effect (NOE, −3.3 ppm). Water peaks (0 ppm) have been removed for clarity. (**b**) Images of amide, amine hydroxyl, MT and water peak areas, acquired noninvasively in an example SW1222 tumor at baseline and post-therapy (24 and 72 hours). Maps of the apparent diffusion coefficient (ADC) from diffusion MRI measurements and the transverse relaxation time (T_2_) are also shown. (**c**) Representative T_2_-weighted (left) and CEST reference images (right).

CEST MRI measurements were represented using Z-spectra, a plot of normalized water signal as a function of saturation frequency offset (Fig. 3a). Our assignment of exchange peaks in Z-spectra is described in the Methods section, and consisted of amide, amine (possibly also containing a contribution from guanidinium) and hydroxyl groups, located at approximately 3.5, 2.4 and 1.2 ppm from water, respectively^27–29^. We also assigned a single, broad peak corresponding to contributions from magnetization transfer (MT, −2.4 ppm^27^), relayed nuclear Overhauser effect (NOE, −3.3 ppm^30^) and lipids (−1 to −5 ppm^29^), which we refer to as NOE/MT. Fitting our summed Lorentzian model on a pixel-by-pixel basis allowed images of each Z-spectrum contribution to be generated (Fig. 3b). The stability of baseline frequency during Z-spectrum acquisition was found to be within 0.1 ppm (see Supplementary Information), and representative T_2_-weighted and CEST reference images are shown in Fig. 3c.

**Figure 4:**
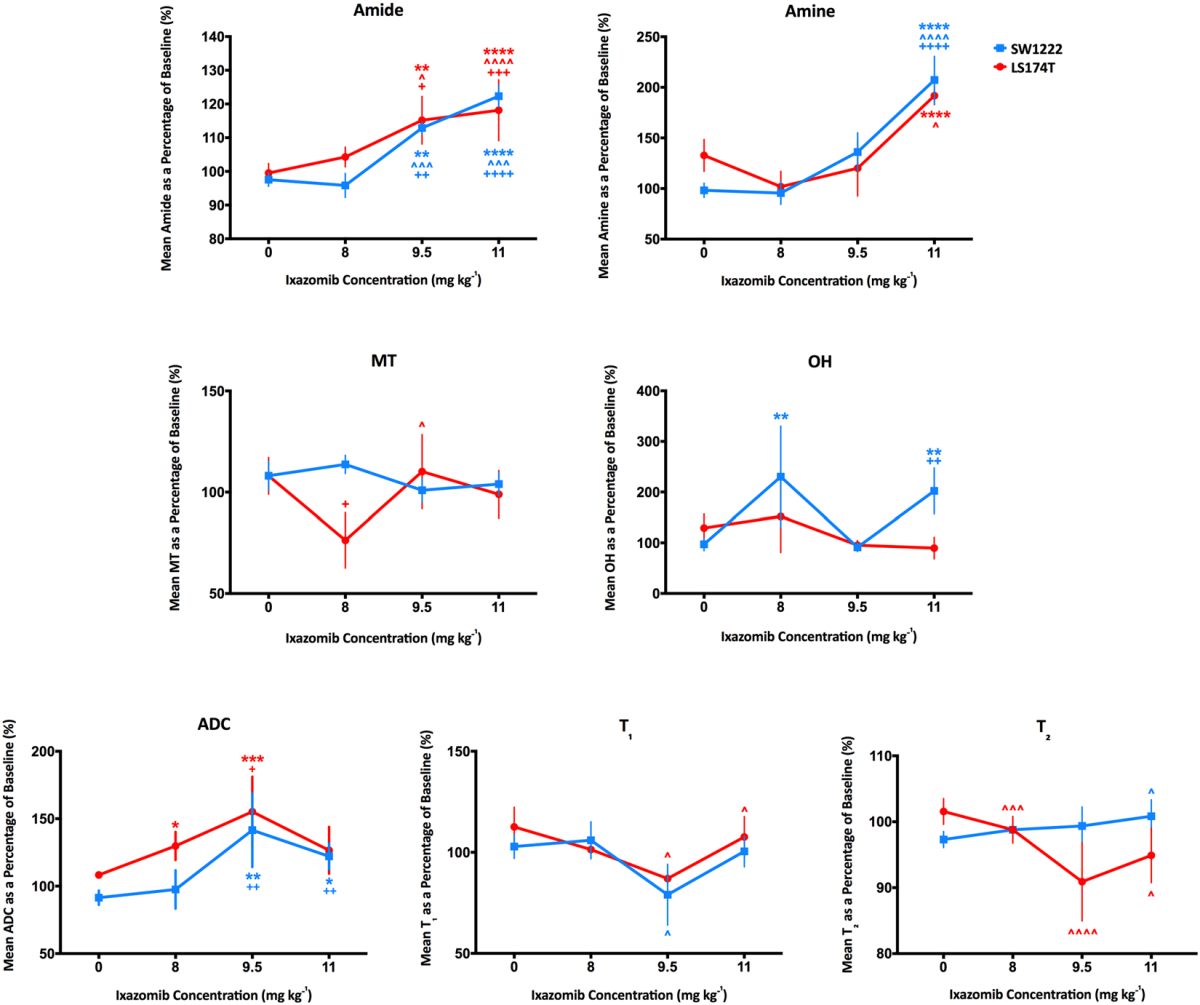
Summary of changes in quantitative, non-invasive MRI parameters, as a function of Ixazomib dose (8, 9.5 and 11 mg kg^−1^), at 72 hours post-dosing. Only CEST measurements associated with amines and amide groups show a correspondence with Ixazomib dose. Significant increases, in a dose-dependent manner, were observed in amine and amide peak areas from CEST MRI measurements. Tumor apparent diffusion coefficient (ADC) also increased significantly for the two highest doses, and T_2_ decreased significantly in LS174T tumors only (the top and bottom set of asterisks or crosses represents LS174T and LS174T, respectively, where two mean values overlap). Data points are mean ± standard error; *P < 0.05, **P < 0.01, ***P < 0.001, ****P < 0.0001 (two-way ANOVA); *compared to baseline, ^^^compared to measurement at 24 hours post-Ixazomib dose, ^+^compared to control.

**Figure 5:**
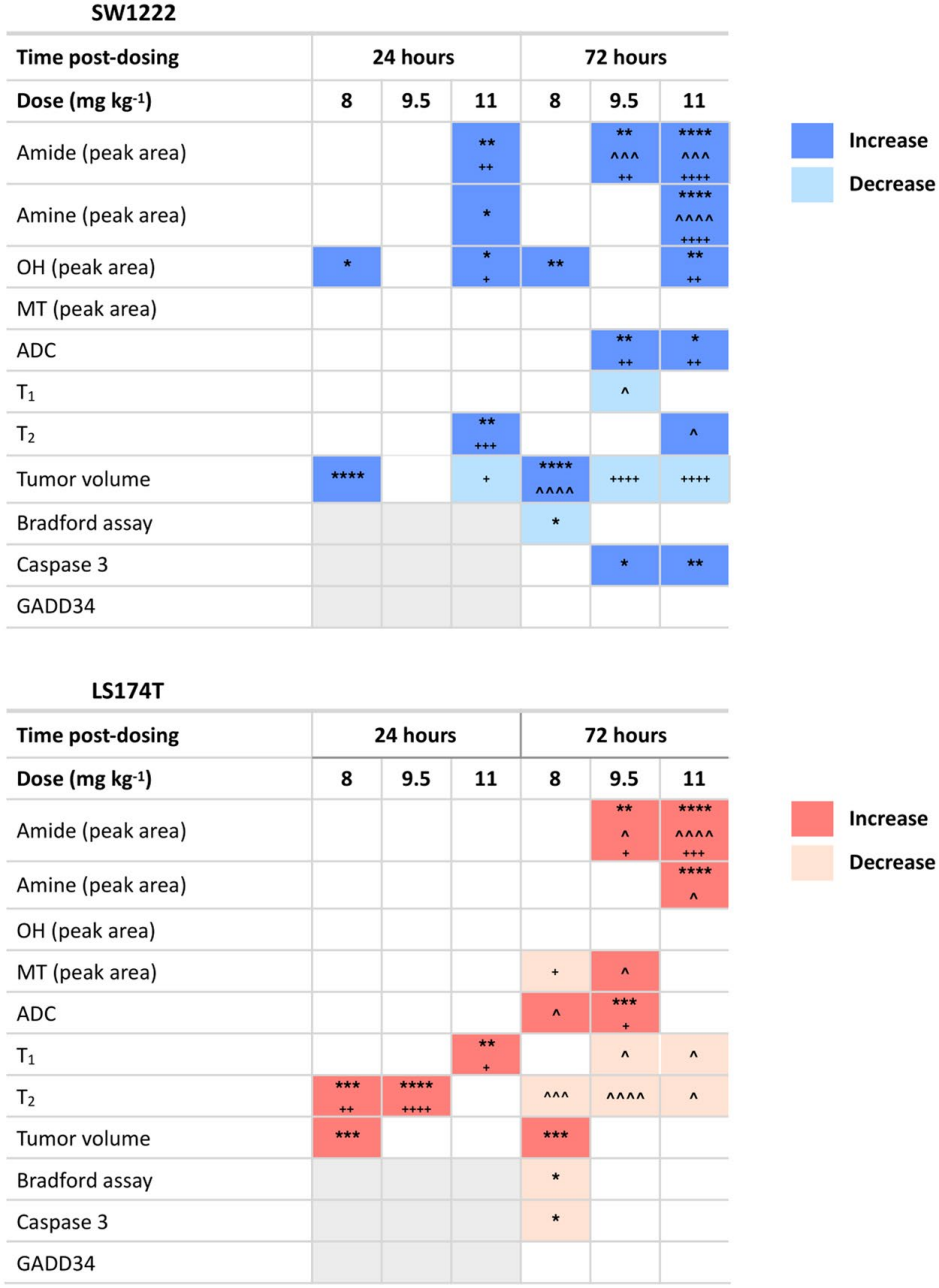
Summary of significant changes (P < 0.05) in each quantitative parameter measured, relative to controls. Dark blue or red cells indicate a significant increase, light blue or red cells indicate a significant decrease, white cells no significant change, and grey cells show where no data were available. *P < 0.05, **P < 0.01, ***P < 0.001, ****P < 0.0001 (two-way ANOVA); *compared to baseline, ^^^compared to measurement at 24 hours post-Ixazomib dose, ^+^compared to control.

The area under amide and amine peaks significantly increased following Ixazomib treatment, relative to pre-treatment measurements (Figs 4 and S1). A summary of all significant changes, for each tumor type, Ixazomib dose and time post-dosing is provided in Fig. 5. These data, in combination, demonstrate the smaller magnitude of change in parameter values following treatment in LS174T tumors compared with SW1222 tumors, potentially reflecting their lower sensitivity to Ixazomib.

### Changes in amide and amine peaks were significantly correlated with Ixazomib dose

A significant correlation was found between amide peak area and Ixazomib dose in SW1222 tumors at 72 hours (r = 0.98, P < 0.05) (Fig. 4). This was also reflected in the change in the amine area of the Z-spectrum, with a strong positive correlation between the dose concentration and amine signals (r = 0.97, P < 0.05). Conversely, no parameters in LS174T tumors exhibited a dose-dependent response, which could reflect their reduced sensitivity to Ixazomib, compared with SW1222 tumors.

### Gross tissue protein measurements from Bradford assay displayed a complex relationship with Ixazomib dose

**Figure 6:**
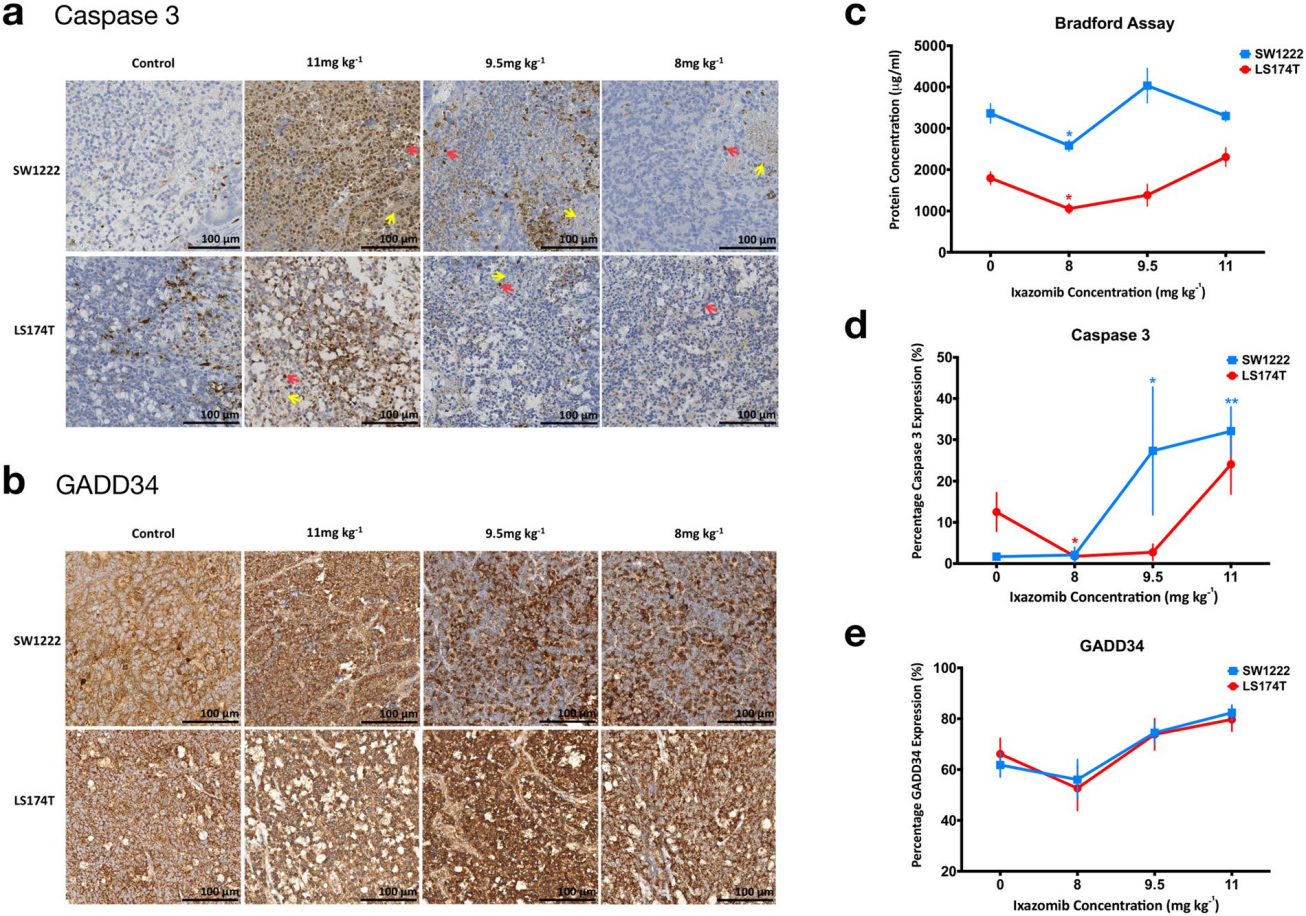
*Ex vivo* analysis of resected tumor tissue. Images of (**a**) caspase 3 and (**b**) GADD34 expression at 72 hours following dosing with vehicle, 8 mg kg^−1^, 9.5 mg kg^−1^ or 11 mg kg^−1^. Caspase 3-positive nuclei are stained brown (as labeled with a red arrow) and blue otherwise (haematoxylin), and example necrotic areas are labeled with a yellow arrow. GADD34-positive areas are stained brown and nuclei are counter-stained in blue with haematoxylin. (**c**) The results of Bradford assay, to measure gross tissue protein. Summary data from quantitative analysis of immunohistochemistry images are shown for caspase 3 (**d**) and GADD34 (**e**), for each study group. The scale bar in (**a** and **b**) represents 100 μm. Data points are mean ± standard error (*P < 0.05, **P < 0.01, ordinary one-way ANOVA of Mann-Whitney test).

Bradford assay at 72 hours showed a significant difference in mean protein concentration between SW1222 and LS174T control tumors (1386 μg ml^−1^, P < 0.001). Both SW1222 and LS174T tumors had a small but significantly lower protein concentration than control tumors when dosed at 8 mg kg^−1^, whereas higher doses did not induce a significant change (Fig. 6c).

No significant correlation was found between Bradford assay results and *in vivo* amide and amine peak area (P > 0.05, Spearman’s rho). Equally, no significant differences were found in baseline CEST parameters, between tumors, which could have reflected the difference found between tumor types in Bradford assay results.

### Diffusion MRI measures reflected changes in CEST parameters, but T_1_ and T_2_ showed mixed changes

A significant increase was found in ADC from baseline, in SW1222 tumors at 72 hours, for Ixazomib doses of 9.5 and 11 mg kg^−1^ (41.5%, P < 0.01 and 22.1%, P < 0.05, respectively), which were significantly greater than control tumors (Figs 4 and 5). The change in ADC from baseline was also significantly correlated with Ixazomib dose (P < 0.01). In LS174T tumors, only the 9.5 mg kg^−1^ group showed a significant increase, when compared against baseline (55.19%, P < 0.001) and control tumors (46.92%, P < 0.05).

T_1_ and T_2_ showed a more mixed set of changes in both tumor types, with fewer time points achieving significance (see Figs 4 and 5). T_1_ and T_2_ showed both increases and decreases over time, and did not display a simple relationship with Ixazomib dose, other than for the change in T_2_ in SW1222 tumors at 72 hours, which was directly correlated with dose (r = 0.99, P < 0.01).

### Immunohistochemical markers of apoptosis significantly correlated with Ixazomib dose in SW1222, but not LS174T tumors

To evaluate the relationship between CEST imaging biomarkers and physiological changes caused by proteasome inhibition, we used immunohistochemistry to measure the percentage expression of cell death factor, caspase 3, and GADD34. Our results revealed significantly higher caspase 3 expression in SW1222 tumors for doses of 9.5 mg kg^−1^ (25.61%, P < 0.05) and 11 mg kg^−1^ (29.87%, P < 0.01), relative to controls (Fig. 6). Caspase 3 expression was also significantly correlated with Ixazomib dose (r = 0.95, P < 0.05). Though small, there was significantly lower caspase 3 expression in LS174T tumors for a dose of 8 mg kg^−1^ (4.57%, P < 0.05), relative to controls, but no significant differences at higher doses. Whilst there was no significant change in GADD34 percentage expression relative to controls in either SW1222 or LS174T tumors (Fig. 6), as for caspase 3, a significant correlation was found between GADD34 expression with increasing dosing concentration in SW1222 tumors (r = 0.96, P < 0.05).

## Discussion

Noninvasive imaging biomarkers for cancer therapy are critically needed in the clinic, particularly those that can detect a functional response prior to much slower changes in tumor volume^24^. CEST is a noninvasive imaging technique, based on MRI, in which image contrast is induced via the exchange of protons between water and chemical groups such as amides, amines and hydroxyls^20^. The presence of amide groups in protein backbones and amine groups on peptides make CEST sensitive to changes in the concentration of both types of molecules^5,31–34^, and thus as a potential biomarker of disrupted protein homeostasis. We therefore aimed to evaluate the change in CEST MRI parameters (alongside other more conventional MRI measures) following dosing with Ixazomib, in two colorectal carcinoma tumor xenograft models (SW1222 and LS174T).

Our experiments showed that Ixazomib elicited a significant growth-inhibitory effect in both colorectal cell lines, as evidenced by a reduction in cell viability *in vitro* and the reduction of tumor growth rates *in vivo*. SW1222 tumors were found to be more sensitive to Ixazomib than LS174T tumors, which could be due to their different K-Ras mutations: SW1222 cells have a K-Ras mutation at A146V^35^, compared with G12D for LS174T tumors^36^, which has been proposed to infer a greater sensitivity to proteasome inhibition^9^. In addition, the microenvironment of the two tumor types could provide a source of resistance *in vivo*, with LS174T tumors being less vascular, less differentiated and less perfused than SW1222 tumors^37–39^.

Our CEST measurements showed a significant increase in amide, amine and hydroxyl peak areas with time following Ixazomib treatment, with the greatest effects observed at 72 hours following dosing. Moreover, several parameters varied in a dose-dependent manner, with a significant correlation measured between amide and amine peak size and Ixazomib dose in SW1222 tumors. This reflected similar trends in our immunohistochemical measurements of caspase 3 and GADD34 expression, which were themselves consistent with previous studies^5,23^. This induction of apoptosis could also explain the observed increases in ADC, in which changes in cell size and the induction of micro-necrosis causes less restricted water diffusion^37^. However, the relationship between CEST parameters and Bradford assay were less straightforward to interpret, with no apparent correspondence evident between the two measurement types. Several factors might help to explain this disparity. Firstly, CEST MRI is sensitive to mobile protein and peptides, whereas the Bradford assay is unaffected by amino acids or peptides smaller than 3 kDa (according to manufacturer’s instructions). Secondly, CEST contrast is dependent on several other parameters including *T*_1_, *T*_2_ and pH, which were not controlled for in this study. *T*_1_ and *T*_2_ were found to show few consistent changes when measured directly, so it is arguable that these were unlikely to have influenced CEST measurements significantly. The pH of tumor tissue could also have influenced our measurements, and a fully quantitative model^27^ could potentially be implemented to separate proton exchange rate and pool sizes. Interestingly, we found evidence that Ixazomib causes a mild stimulatory effect at low doses^40^. This bi-phasic effect has been previously observed in a wide range of anticancer agents^40–42^ alongside physical interventions such as radiotherapy^43^. *In vitro* MTT assays revealed increased cell viability at low doses, and tumor growth was slightly enhanced at an *in vivo* dose of 8 mg kg^−1^. Likewise, caspase 3 expression was slightly decreased, relative to controls, in LS174T tumors dosed at 8 mg kg^−1^. Whether this effect was reflected in MRI measurements is unclear, as no significant changes were observed in CEST parameters (amide and amine areas) at low Ixazomib doses. However, this effect warrants further investigation, particularly if the treatment of tumors with poor delivery profiles (for example, pancreatic adenocarcinoma) is currently under consideration, as delivered doses might be significantly lower than expected. In this context, in particular, amide and amine CEST signals and ADC could find strong utility, alongside complementary measures of blood flow and interstitial transport.

A limited number of studies have previously identified CEST MRI as a potential biomarker of response to anti-cancer therapies. Sagiyama *et al*. demonstrated that amide signal proportionally decreased with Ki67 (a marker of cell proliferation) in brain tumor models following temozolomide (TMZ) treatment^44^, and the authors suggested that CEST MRI be used for the non-invasive detection of therapeutic response. Another study suggested the biomarker potential of CEST MRI in high intensity focused ultrasound (HIFU) treatment, after observing reduced amide signals in HIFU-treated animal models^45^. Both of these studies demonstrated a decrease in amide signal following therapy, which contrasts with our observation of an increase. The cause of this is possibly due to the different analysis approach used in the current study; in conventional CEST MRI asymmetry analysis, the portion of the Z-spectrum with a frequency greater than water is subtracted from the portion with a frequency lower than water, to produce a magnetization transfer asymmetry (MTR_asym_) spectrum, which forms the basis of further spectral analysis^20^.

In the current study, individual exchange peaks were individually fitted, allowing the influence of amide, amine and hydroxyl groups, alongside NOE and MT effects, to be isolated. However, it is possible that other chemical groups could also have contributed to these peaks; for example, guanidinium groups have been proposed to contribute an exchange peak at ~2 ppm, which could overlap with our assigned amide peak^46,47^. Likewise, we chose to combine MT and NOE effects into a single, broad peak, as this was found to provide greater model stability than fitting both influences independently.

A novel Bayesian summed Lorentzian model enabled semi-quantitative analysis of the CEST data in this study. This model is useful as it provides a straightforward and precise characterization of the signal, but also suffers from some limitations. For example, it is phenomenological in nature, and parameter estimates are specific to the B_1_ power used (3 μT RMS, here). Moreover, the coalescence of exchange and water peaks cannot be easily resolved, which could have affected the quantification of hydroxyl peaks, in particular, and so these data should be treated with more caution than those associated with peaks further removed from water (such as amides). To account for these effects would require a more complex, parametric exchange model (for example, the Bayesian model proposed by Chappell *et al*.^27^. This approach would also enable a more rigorous evaluation of T_1_ changes that were concomitant with changes in other parameters, beyond the bulk T_1_ measurements undertaken here.

In the current study, individual exchange peaks were individually fitted, allowing the influence of amide, amine and hydroxyl groups to be isolated. A limitation to our model was the combining of NOE and MT effects into a single Lorentzian peak, which was determined to provide greater model stability than two separate Lorentzians during our initial model development. However, MT could most likely be modeled as a constant offset in future analyses, due to its broad line shape, thereby allowing MT effects to be modeled independently.

A key question is whether these findings can be translated and reproduced in humans. Clinical MRI is typically undertaken at field strength of 3T, which is lower than the 9.4T system used here. Translation between field strengths presents several challenges (such as reduced separation between exchange peaks, lower signal-to-noise ratios, and slower relaxation rates), but which have been overcome in other translational studies^24,48^. Equally, the evaluation of Ixazomib in human solid tumors is at an early stage and the data presented here demonstrates its efficacy in two colorectal carcinoma xenograft models. It also identifies several promising, noninvasive biomarkers (amide and amine CEST signals and ADC) that could be explored for determining early treatment response and detecting disrupted protein homeostasis. In addition, they could be further evaluated in the assessment of plasma cell malignancies (currently the predominant targets of Ixazomib), with a view to reducing the need for invasive bone marrow aspirates or biopsy.

## Materials and Methods

### Cell culture

SW1222 and LS174T cells were cultured in minimum essential medium eagle (MEME, M4655, Sigma-Aldrich Cell Culture, UK) with 5% fetal bovine serum. On the day of inoculation, the cells were harvested by washing once in phosphate buffered saline (PBS) solution (Sigma-Aldrich Cell Culture, UK) and trypsinised at 37 °C for 5 minutes. The cells were pelleted by suspending in MEME and centrifuged at 1600 × g for 5 minutes. The suspension mixture was removed and the pelleted cells were washed in PBS and then in serum-free media at 1600 × g for 5 minutes. Before the final wash in serum-free medium, 10 µL of the cell suspension mixed with trypan blue (Invitrogen, Thermo Fisher Scientific, USA) was transferred to a cell counting chamber slide (Invitrogen, Thermo Fisher Scientific, USA) and cells were counted with a Countess Automated Cell Counter (Invitrogen, Thermo Fisher Scientific, USA). The cells pelleted after the final wash were suspended in known volume serum free-media.

### *In vitro* MTT assay

MTT (3-(4,5-dimethylthiazol-2-yl)-2,5-diphenyltetrazolium bromide) (Vybrant® MTT Cell Proliferation Assay Kit, Life Technologies, Thermo Fisher Scientific, USA) reagent at 12 mM was prepared according to manufacturer’s instructions. Before treatment, 10,000 cells were seeded in 100 µL of complete media per well, for 9 hours, in a clear polystyrene 96-well flat bottom microplate (Greiner Bio-One, Austria).

Ixazomib was diluted in complete media from 400 to 1.6 nM using 2-fold serial dilution. Media in each well was removed and replaced with 90 µL of Ixazomib, with corresponding concentration. Cells were left for 24, 48 or 72 hours, then labeled with 10 µL of the MTT reagent, and their absorbance were measured, according to instructions (see supplemental methods).

Cell viability was estimated from the absorbance measure (unitless) as a percentage of the mean absorbance of control cells. These data were fitted to the modified Hill equation, from which the *EC*_50_ (half-maximal stimulatory concentration) and *IC*_50_ (half-maximal inhibitory concentration) were estimated (see supplemental methods for details).

### Animal models

All *in vivo* experiments were performed in accordance with the UK Home Office Animals Scientific Procedures Act, 1986 and United Kingdom Coordinating Committee on Cancer Research (UKCCCR) guidelines^49^. All experimental protocols were approved by the University College London Animal Ethics Committee. Mice had access to food and water ad libitum. 5 × 10^6^ SW1222 or LS174T cells were subcutaneously inoculated (0.1 mL per injection) on the lower right flank of 6–8 weeks old female CD1 nu/nu mice (Charles River Laboratories, UK).

### *In vivo* experimental design

Tumors were allowed to grow for 14–16 days, then randomly assigned to treatment and control groups. The treatment group received a single dose of Ixazomib i.v., at 8, 9.5 or 11 mg kg^−1^ (n = 6, 5, 14, respectively). The control group (n = 5, 5, 7 for the treated 8, 9.5 and 11 mg kg^−1^, respectively; total n = 17) received vehicle, consisting of the drug stock solution (2-Hydroxypropyl-β-cyclodextrin, Sigma Aldrich, USA). Treated mice were maintained in a warmed cage using a thermostatic heating pad (Physitemp Instruments, Inc., USA) and an infrared heating lamp (Zoo Med, UK) providing an ambient temperature of 24–26 °C. Hydrogel was provided to prevent dehydration. Tumor volume (*V*) was measured every 2 days using electronic calipers, according to^50^ *V* = *w*^2^*l*/2, where *l* and *w* are the maximal tumor diameter and the diameter orthogonal to this measurement, respectively. The mass of each mouse was also measured using an electric balance (C-MAG HS 7 IKAMAG®, Germany).

Baseline MRI data were acquired 24 hours before drug administration (0 hours), with follow-up sessions at 48 and 72 hours. Immediately following the final MRI scan, mice were culled via cervical dislocation, tumors were resected and sent for *ex vivo* analysis.

### *In vivo* MRI protocol

MRI data were acquired using a 9.4 T scanner (Agilent, USA) with a 39 mm birdcage coil (Rapid MR International, USA). Mice were anaesthetized prior to and throughout each scanning session using isoflurane in O_2_ (2.5% for induction, 1.25–1.75% for maintenance). Core body temperature was maintained 37 °C using a warm water heating system. Ventilation rate was monitored using a respiration pad (SA Instruments, USA) and maintained at 60–80 breaths per minute by adjustment of isoflurane concentration.

Following shimming, a T_2_-weighted, fast spin echo sequence was used for tumor localization and volume measurement (see supplemental methods for details).

#### CEST MRI

Our CEST sequence was based on a gradient echo acquisition with the following parameters: repetition time (TR), 162 ms; echo time (TE), 2 ms; RMS saturation power, B_1,rms_, 3 µT; flip angle, 20°; slice thickness, 1 mm (single slice); matrix size, 64 × 64; FOV, 30 × 30 mm^2^, acquisition time, 8 minutes. The slice was positioned to encompass the largest cross-sectional area of each tumor. Sampling of each line of k-space was preceded by a train of three 50 ms Gaussian pulses to induce saturation, with no dummy scans. We have previously shown that 100 saturation pulses, applied within the outer third of k-space, enable a steady state to be reached^28^. Each pulse had a flip angle of 20°, duration of 2 ms and with 2 ms spacing^28^. Crusher gradients were applied between pulses to spoil any residual transverse magnetization. The sequence was repeated with saturation frequency offsets ranging from −6 to 6 ppm, at intervals of 0.12 ppm. A reference image was also acquired at an offset of 8000 ppm (*S*_0_).

During post-processing, Z-spectra were produced on a pixel-by-pixel basis, according to *Z*(*f*) = *S*(*f*)/S_0_, where *f* is the saturation offset frequency. These data were fitted with a summed Lorentzian model^51^, with peak offsets corresponding to proton exchange between water and amide, amine (plus a potential contribution from guanidinium groups) and hydroxyl groups, alongside a broad peak corresponding to magnetization transfer, nuclear Overhauser effect (NOE) and lipids. Details of this modeling step are provided in the supplemental methods.

#### Diffusion MRI and relaxometry

Full details of diffusion MRI, T_1_ and T_2_ acquisition and quantification procedures are provided in the supplemental materials. In brief, diffusion MRI data were acquired using a multi-slice fast spin-echo sequence, with b-values ranging from 150 to 1070 s mm^−2^ and acquisition time of 6 minutes. The apparent diffusion coefficient was quantified from these data by fitting to a simple exponential function (equ. 5). T_1_ and T_2_ were estimated from data acquired with a Look-Locker^52^ segmented inversion recovery sequence and a multi-echo multi-slice spin-echo sequence (acquisition time of 7 and 4 minutes, respectively).

### *Ex vivo* analysis of resected tumor samples

Resected tumors were cut in half: one half was flash-frozen in liquid nitrogen and stored at −80 °C (for Bradford Assay) and the other half was fixed in 5% formalin for immunohistochemical analysis.

#### Bradford Assay

Tumor protein was extracted (T-PER® Tissue Protein Extraction Reagent, Thermo Fisher Scientific, USA) and Bradford assays were carried out (Pierce Coomassie Bradford Protein Assay Kit, Thermo Fisher Scientific, USA) according to manufacturer’s instructions.

#### Immunohistochemistry

GADD34 and cleaved caspase-3 immunohistochemistry was performed on all tumor samples. All slides were haematoxylin counterstained and digitalized (LEICA SCN400, LEICA Microsystems, UK). Slides were analyzed and quantified via user-defined algorithms using Definiens Tissue Studio and Developer (Definiens AG, Germany). Percentage expression of each marker was measured from mean of percentage positive tissue area and percentage positive nuclei. Details of staining, analysis and quantification are provided in the supplemental methods.

### Statistical analysis

Data were aggregated from pixel data in each tumor, and their median value used as a measure of central tendency in subsequent analysis (rather than mean values, in order to limit the influence of extreme or outlier values resulting from convergence at local minima). Longitudinal data were normalised to the baseline measurement and expressed as a percentage change. Two-way analysis of variance between groups (ANOVA) (GraphPad Prism X.6.0.1, USA) was used for statistical analysis. Both the difference between control and treated groups at the same time points (between group analysis), and the difference between post-treatment and baseline data (within group analysis) were tested for statistical significance. P < 0.05 was considered significant. Correlations were assessed using Spearman’s rho.

## Electronic supplementary material

Supplementary Information

## Data Availability

The datasets generated during and/or analysed during the current study are available from the corresponding author on reasonable request.
