# Supplementary material for: Non-invasive imaging of disrupted protein homeostasis induced by proteasome inhibitor treatment using chemical exchange saturation transfer MRI
Source: Sci Rep. 2018 Oct 10;8:15068. doi: 10.1038/s41598-018-33549-x (PMC6180115; doi:10.1038/s41598-018-33549-x)
Supplement: Supplementary file 1 — Supplementary Information [file 41598_2018_33549_MOESM1_ESM.docx]

**Supplementary Information**

**Non-invasive imaging of disrupted protein homeostasis induced by proteasome inhibitor treatment using chemical exchange saturation transfer MRI**

**Yanan Zhu^1^, Rajiv Ramasawmy^1^, Sean Peter Johnson^1^, Valerie Taylor^1^, Alasdair Gibb^2^, R Barbara Pedley^3^, Nibedita Chattopadhyay^4^, Mark F Lythgoe^1^, Xavier Golay^5^, Daniel Bradley^6^, and Simon Walker-Samuel^1,*^**

^1^UCL Centre for Advanced Biomedical Imaging, Division of Medicine, University College London, London WC1E 6DD, United Kingdom

^2^Neuroscience, Physiology & Pharmacology, University College London, London WC1E 6BT, United Kingdom

^3^UCL Cancer Institute, University College London WC1E 6DD, United Kingdom

^4^Cancer Pharmacology, Takeda Pharmaceutical International Corporation, Cambridge, MA 02139, United States

^5^Institute of Neurology, University College London, London WC1N 3BG, United Kingdom

^6^Biomedical Imaging Group, Takeda Pharmaceutical International Corporation, Cambridge, MA 02139, United States

***Corresponding author**

Simon Walker-Samuel

Centre for Advanced Biomedical Imaging, University College London

Lower Ground Floor, Paul O’Gorman Building, 72 Huntley Street,

London, WC1E 6DD

Tel.: +44 20 7679 6329

Email: simon.walkersamuel@ucl.ac.uk

**Supplementary Figure**

**Figure S1.** Graphs showing the relationship between the relative change in each non-invasive imaging parameter and time, for three different doses of Ixazomib (8, 9.5 and 11 mg kg^-1^) and vehicle, in (I) SW1222 and (II) LS174T tumors. Imaging parameters shown are: (a) median amide area, (b) median amine area, (c) median MT area, (d) median OH area, (e) median ADC, (f) median volume, (g) median T_1_ and (h) median T_2_. Error bars correspond to the standard error of the mean (*P<0.05, ** P<0.01, *** P<0.001, **** P<0.0001; * = compared to baseline, ^ = compared to measurement at 24 hours after Ixazomib treatment, ^+^ = compared to control).

**Supplemental Methods**

**MTT assay**

**Labelling.** Ixazomib was diluted from 10 mM stock solution in complete media from 400 to 0.390 nM using 2-fold serial dilution. Media in each well was removed and replaced with 90 µL of Ixazomib, with corresponding concentration. Cells were left for 24, 48 or 72 hours, then labelled with 10 µL of the 12 mM MTT reagent (Vybrant® MTT Cell Proliferation Assay Kit, Life Technologies, Thermo Fisher Scientific, USA), and incubated for 2 hours at 37°C. After the incubation period, the media was removed and replaced with 100 µL of DMSO per well, and mixed thoroughly using an orbital plate shaker for 2 minutes. The microplate was incubated for 37°C for 10 min and were read using a microplate absorbance reader at 540 nm.

**Quantification**. We estimated cell viability from the absorbance measure (unitless), in which the viability of treated cells was expressed as a percentage of the mean absorbance of control cells. Mean measurements of cell viability, expressed as a percentage change from baseline, were fitted to the modified Hill equation:

| $y= m+\frac{p_{1}-m}{\left( 1+\frac{{EC}_{50}}{\left[ I \right]} \right)^{nH_{1}}}+ \frac{p_{2}-m}{\left( 1+\frac{\left[ I \right]}{{IC}_{50}} \right)^{nH_{2}}}$ | [1] |
| --- | --- |

where *m* is the maximum percentage viability; *p_1_* is the viability at low concentration (constrained to 100%), and *p_2_* is the viability at higher concentrations; [*I*] is the concentration of Ixazomib (nM); *EC_50_* and *IC_50_* are concentrations that produced half-maximal stimulatory and inhibitory effects (nM), respectively; *nH_1_ and nH_2_* are unitless Hill factors for stimulatory and inhibitory slopes, respectively.

The modified Hill equation describes the binding of a drug to a receptor, with an empirical modification factor (*nH*) [^1^](#_ENREF_1). In our implementation, we modelled a single Ixazomib-binding site on the proteasome. The relationship between binding the proteasome and inhibition of cell proliferation is unknown. The data were fitted with a two-component Hill Equation to estimate the *IC_50_* concentration and maximum effect. Data were fitted by using Microsoft Excel Solver function. *IC_50_,*  Hill coefficients and plateau parameters were fit to minimise the sum of squares independently for each data set.

**Immunohistochemistry**

**Staining and Imaging**. GADD34 and cleaved caspase-3 immunohistochemistry was performed using a Ventana Discovery XT, with the Ventana DAB Map Kit (760-124, Ventana Medical Systems, Inc, USA). Details of the protocols used are provided in supplemental methods. Heat induced epitope retrieval was performed using an EDTA buffer (pH8.0). Anti-GADD34 (SC-8327, Santa Cruz Biotechnology, USA) primary antibody incubation was for 4 hours using a 1:50 dilution, followed by amplification with the Ventana Amplification Kit (760-080, Ventana Medical Systems, Inc, USA). Anti-Cleaved-Caspase-3 (9661L, Cell Signaling Technology, The Netherlands) primary antibody incubation was for 30 minutes using a 1:100 dilution. Swine anti-Rabbit (E0353, Dako, Agilent, USA) secondary antibody incubation was for 30 minutes, using a 1:200 dilution. All slides were haematoxylin counterstained and digitalized using LEICA SCN400 scanner (LEICA Microsystems, UK), with ×40 magnification, 65% image compression.

**Analysis and quantification.** Slides were analyzed and quantified using Definiens Tissue Studio and Developer (Definiens AG, Germany). Marker expression was quantified by measuring percentage area and percentage nuclei of the positive regions. For the percentage area calculation, distinctive regions of a tumor tissue slide were first manually classified from a sample slide. A typical immunostained tumor tissue was classified into five regions: positive, viable, necrotic, dermis/fat, and glass, in the training process of Tissue Studio composer actions. The software then automatically identified and segmented each slide into five regions of interest (ROI) with threshold based on the intensity of haematoxylin (blue) and chromogen (brown). After the automatic segmentation of all slides was completed, the slides were reassessed manually by the user to correct any mismatched regions (in ‘correction mode’). Automatic quantification was then carried out, where the absolute area and percentage area of each ROI over total tissue was calculated, where the total tissue area was the sum of positive, viable and necrotic areas. For the percentage nuclei quantification, in the software training process, tumor tissue area (from segmentation described above) were classified as ‘nuclei’ or ‘cytoplasm’, and within the ‘nuclei’, they were further classified into ‘positive’, ‘viable’ and ‘necrotic’. The tissues were automatically labelled based on the preset threshold values of the stain intensity. The absolute number and percentage of each class of nuclei were automatically quantified, where the total of nuclei were the sum of positive, viable and necrotic nuclei. The percentage expression of caspase 3 and GADD34 were calculated by taking a mean value between the percentage area and percentage nuclei.

**CEST Z-spectrum model**

The Lorentzian function for the *i^th^* pool was given by [^2^](#_ENREF_2):

|  | ${L(f)}_{i}=\frac{c_{i}}{\pi}\left[ \frac{b_{i}}{{(f-a_{i}-f_{0})}^{2}+b_{i}^{2}} \right]$ | [3] |
| --- | --- | --- |

which were fitted to Z-spectra according to:

|  | $Z\left( f \right)=1-\sum_{i=0}^{n-1} {L(f)}_{i}$ | [4] |
| --- | --- | --- |

*where a_i_* is the estimated frequency offset of the *i^th^* pool, *b_i_* is the width parameter, *c_i_* is a scaling factor and *f*_0_ is a global frequency offset to account for inhomogeneities in the static magnetic field (*B*_0_). Bayesian maximum a posteriori was used to fit *a, b,* *c* and *f*_0_ to Z-spectra [^3^](#_ENREF_3), using the fminunc minimization algorithm in Matlab (MathWorks, USA) with the exception of a_0_ (water pool), which was fixed at 0 ppm. Gaussian prior distributions for each parameter were defined as shown in **Table S1,** which were derived from Chappell *et al*. [^4^](#_ENREF_4). The area under each peak was used to characterise each pool in subsequent analysis.

| **Pool (index)** | **Gaussian prior parameters: mean (s.d.)** | | |
| --- | --- | --- | --- |
|  | ***b* (ms)** | ***a* (ppm)** | ***f_0_* (ppm)** |
| Water (0) | 5 (3) | n/a | 0 (3) |
| Amide (1) | 8 (5) | 3.5 (0.6) |  |
| Amine (2) | 8 (5) | 2.4 (0.6) |  |
| Hydroxyl (3) | 5 (5) | 1.2 (0.6) |  |
| MT & NOE (4) | 0.1 (1) | -2.4 (0.6) |  |

**Table S1.** Gaussian prior distribution parameter values for the maximum a posteriori model, expressed as mean (s.d.) (derived from Chappell *et al*. [^4^](#_ENREF_4)). Entries marked with ‘n/a’ were fixed (see text for details).

**Tumor volume measurement with MRI**

A T_2_-weighted, fast spin echo sequence was used for tumor localization and tumor volume measurements, which included the following parameters: repetition time (TR), 1.5s; echo train length, 4; effective echo time (TE_eff_), 17.2 ms; slice thickness, 1mm; number of slices, 20; number of averages, 4; matrix size, 128 × 128; field of view (FOV), 30 × 30 mm^2^; acquisition time, 5-10 minutes. Regions of interest (ROIs) were drawn, covering the entire tumor mass (Matlab). The number of pixels in these volumetric ROIs was multiplied by the voxel volume (238×238×1000 µm^3^) to estimate tumor volume.

**Diffusion MRI acquisition and post-processing**

A multi-slice diffusion-weighted fast spin echo sequence was used which included the following parameters: TR, 1500 ms; TE_eff_, 2000 ms; b-value, 150, 300, 503, 760, 1070 s mm^-2^; slice thickness, 1 mm; number of slices, 8; matrix size, 128 × 128; FOV, 30 × 30 mm^2^; acquisition time, 6 minutes. The apparent diffusion coefficient (ADC) was quantified from these data by fitting a single exponential to signal intensity values, of the form:

|  | $S\left( b \right)=S_{0}exp(-ADC.b)$ | [5] |
| --- | --- | --- |

where *S*_0_ and ADC are fitted parameters and *b* is the b-value. This was undertaken on a pixel-by-pixel basis, and pixels were fitted using the maximum a posteriori algorithm that took into account the Rician noise distribution [^5^](#_ENREF_5). See **Figure S2** for an example fit to data from an LS174T tumor.

***T*_1_ measurement**

A Look-Locker segmented inversion recovery sequence [^6^](#_ENREF_6) was used to estimate the longitudinal relaxation time, *T*_1_, with the following parameters: number of inversion points, 50; TR, 110 ms; TE, 1.18 ms; slice thickness, 1mm; number of slices, 8; matrix size, 128 × 128; FOV, 30 × 30 mm^2^; acquisition time, 7 minutes; inversion time spacing, 110 ms; repetition time between Look-Locker sampling pulses, 2.3 ms; 50 inversion recovery readouts; 4 lines per segmented acquisition. In order to sample the longitudinal magnetization during its recovery following an inversion pulse, the Look-Locker sequence applies a train of small flip angle readout pulses, separated by a fixed inversion time (TI). This pulse train partially saturates the signal, resulting in an apparent longitudinal relaxation time, *T*_1_*, shorter than the true *T*_1_ time. *T*_1_* was estimated by fitting data to the following three parameter model:

|  | $S\left( b \right)=S_{0}\left( 1-\beta exp\left( {-TI}/{T_{1}^{*}} \right) \right)$ | [6] |
| --- | --- | --- |

*T*_1_ was then estimated according to [^7^](#_ENREF_7):

|  | $T_{1}=T_{1}^{*}\left( \beta-1 \right)$ | [7] |
| --- | --- | --- |

See **Figure S2** for an example fit to data from an LS174T tumor.

***T*_2_ measurement**

A multi-echo multi-slice imaging sequence was used to estimate *T*_2_, using the following parameters: TR, 1500 ms; 16 echo times (TE) ranging from 8 to 128 ms, at 8 ms intervals; slice thickness, 1 mm; number of slices, 8; matrix size, 128 × 128; FOV, 30 × 30 mm^2^; acquisition time, 4 minutes. *T*_2_ and *S*_0_ values were estimated using the following equation:

|  | $S\left( TE \right)=S_{0}exp(-{TE}/{T_{2}})$ | [8] |
| --- | --- | --- |

where S_0_ and *T*_2_ are fitted parameters. Again, the maximum a posteriori algorithm was used, which took into account the Rician noise distribution of MRI magnitude data [^3^](#_ENREF_3). See **Figure S2** for an example fit to data from an LS174T tumor.


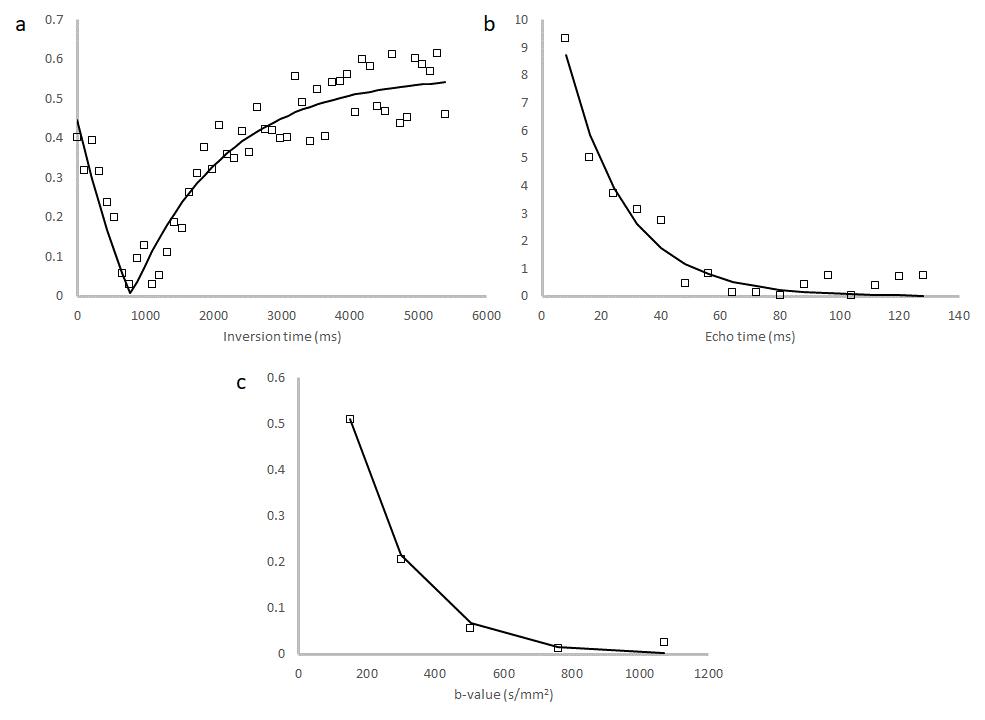


**Figure S2.** Example fits to (a) segmented Look-Locker, (b) multi-echo, and (c) diffusion-weighted data acquired from an LS174T tumor. Values on the vertical axis are arbitrary signal.

**CEST acquisition stability**

To evaluate the stability of the CEST acquisition sequence, Z-spectra were acquired in three murine subcutaneous tumors (LS174T), which were established according to the procedures described in the main manuscript. The CEST sequence was modified, such that the offset frequency was fixed at the reference frequency (8000 ppm), in an acquisition that was otherwise identical to the standard CEST acquisition. ROIs corresponding to the tumor were defined during post-processing in each set of data, and the change in signal phase, relative to the first acquisition, was measured in each pixel. These data were averaged and plotted against time.

The results of this experiment are shown in **Figure S3**, which reveals a variation in baseline frequency ranging between ±0.1 ppm, with no clear trend with time. This variability is less than the spectral spacing of saturation frequencies in the standard CEST acquisition (0.12 ppm), and there were no significant changes in frequency (MANOVA, P>0.05). This suggests that the acquisition sequence was stable, in regard to baseline frequency shift, during the course of a standard Z-spectrum acquisition.


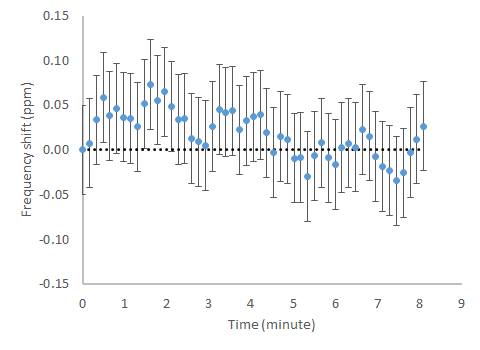


**Figure S3.** Frequency shift as a function of time, for a CEST acquisition with saturation offset fixed at the reference frequency (8000 ppm). The data were acquired in three subcutaneous murine tumors (LS174T) and averaged. Error bars represent the standard error in the mean.

**References**

1 Prinz, H. Hill coefficients, dose-response curves and allosteric mechanisms. *J Chem Biol* **3**, 37-44, doi:10.1007/s12154-009-0029-3 (2010).

2 Desmond, K. L., Moosvi, F. & Stanisz, G. J. Mapping of amide, amine, and aliphatic peaks in the CEST spectra of murine xenografts at 7 T. *Magn Reson Med* **71**, 1841-1853, doi:10.1002/mrm.24822 (2014).

3 Walker-Samuel, S. *et al.* Bayesian estimation of changes in transverse relaxation rates. *Magn Reson Med* **64**, 914-921, doi:10.1002/mrm.22478 (2010).

4 Chappell, M. A. *et al.* Quantitative Bayesian model-based analysis of amide proton transfer MRI. *Magn Reson Med* **70**, 556-567, doi:10.1002/mrm.24474 (2013).

5 Walker-Samuel, S., Orton, M., McPhail, L. D. & Robinson, S. P. Robust estimation of the apparent diffusion coefficient (ADC) in heterogeneous solid tumors. *Magn Reson Med* **62**, 420-429, doi:10.1002/mrm.22014 (2009).

6 Look, D. C. & Locker, R. D. Time saving in measurement of NMR and EPR relaxation Times. *Rev Sci Instrum* **41**, 250-251 (1970).

7 Deichmann, R. & Haase, A. Quantification of T1 Values by SNAPSHOT-FLASH NMR Imaging. *J Magn Reson* **96**, 608-612, doi:10.1016/0022-2364(92)90347-A (1992).
